# Efficacy of Laser‐Assisted Intralesional Triamcinolone for Pathological Scars: A Systematic Review and Meta‐Analysis

**DOI:** 10.1111/jocd.71064

**Published:** 2026-08-17

**Authors:** Bader N. Almutawaa, Kholoud Almahzoum, Noof Albannai, Faisal Fahad Alsuwailem, Shaan Alghanim, Danah Jamil Hammadi, Aljazi Jassim Alkhazal, Fawaz A. Al‐Abdulkareem, Malak A. Alshamali, Abdulmuhsin H. Al‐Rashid, Shouq Alkhatlan, Abdullah M. Alharran, Waleed Alfailakawi

**Affiliations:** ^1^ Kuwait Institute for Medical Specialization Kuwait City, Al Asimah Kuwait; ^2^ Department of General Surgery Sheikh Jaber Al‐Ahmad Al‐Sabah Hospital, Ministry of Health Kuwait City Kuwait; ^3^ School of Medicine Trinity College Dublin Dublin Ireland; ^4^ College of Medicine Alexandria University Alexandria Egypt; ^5^ Royal College of Surgeons in Ireland Dublin Ireland; ^6^ School of Medicine, Royal College of Surgeons in Ireland Medical University of Bahrain (RCSI‐MUB) Busaiteen Bahrain; ^7^ University College Dublin Dublin Ireland; ^8^ Faculty of Medicine Kuwait University Kuwait City Kuwait; ^9^ College of Medicine and Medical Sciences Arabian Gulf University Manama Bahrain

**Keywords:** hypertrophic scar, intralesional injection, keloid, laser therapy, scar revision, triamcinolone acetonide

## Abstract

**Background:**

Pathological scars result from aberrant wound healing and remain challenging to treat. Intralesional triamcinolone acetonide is the current mainstay of therapy. Recently, adjuvant laser therapy has emerged as a promising strategy to enhance drug penetration and modulate scar vasculature and collagen remodeling. This meta‐analysis assesses the clinical efficacy of laser‐assisted intralesional triamcinolone in the treatment of pathological scars.

**Methods:**

In March 2026, we conducted a systematic search of PubMed, Scopus, Web of Science, Embase, and the Cochrane Library for randomized controlled trials (RCTs) and non‐randomized studies directly assessing laser plus intralesional triamcinolone. For the meta‐analysis, we used R 4.5.0 with R Studio 2024.12.1 + 563.

**Results:**

Our review included five RCTs and three non‐randomized comparative studies. Six studies encompassing 248 patients were included in the meta‐analysis. Compared with intralesional triamcinolone alone, laser combination therapy showed no significant difference in VSS/mVSS reduction (SMD = 0.28, 95% CI: −0.53 to 1.10, *p* = 0.496), with substantial heterogeneity (I^2^ = 83.1%). However, when compared with laser monotherapy, the combination significantly improved VSS/mVSS (SMD = −1.16, 95% CI: −1.85 to −0.48, *p* = 0.0008; I^2^ = 16.9%). Patient satisfaction appeared higher with combination therapy, but certainty was low, and the comparison with laser monotherapy was based on a single study. For VSS subscales vs. laser alone, combination therapy significantly improved vascularity, pliability, and height, but not pigmentation. Recurrence rates did not differ significantly between groups.

**Conclusion:**

Laser plus intralesional triamcinolone may improve scar scores and patient satisfaction compared with laser monotherapy, but current evidence does not demonstrate superiority over intralesional triamcinolone alone. Recurrence benefit remains uncertain. However, our findings are limited by the small number of RCTs and substantial heterogeneity. Future adequately powered RCTs with longer follow‐up periods are warranted.

## 1 Introduction

Abnormal wound healing can lead to pathological scar formation, placing a considerable burden on patients worldwide [1]. Keloids and hypertrophic scars, along with post‐burn contractures, are linked to functional limitation and psychological distress that go far beyond cosmetic concern [1, 2]. These fibroproliferative lesions arise from disordered wound‐healing pathways marked by excessive collagen accumulation and abnormal extracellular matrix turnover, largely driven by dysregulated transforming growth factor‐beta (TGF‐β) signaling and persistent fibroblast activation [3, 4].

The prevalence of pathological scarring varies considerably across populations, with greater susceptibility observed in individuals with darker skin phototypes, those with a hereditary predisposition, and patients who sustain severe thermal or traumatic injury [5]. Although this area has been investigated for many years, no single treatment has yet achieved wide acceptance as a consistently effective option for preventing or reversing established pathological scars [6].

Single‐modality approaches, including intralesional corticosteroid injections, laser ablation, and other isolated interventions, often provide only limited benefit and are accompanied by inconsistent recurrence rates. Triamcinolone acetonide remains the usual first‐line intervention, yet reported response rates range from 50% to 100%, and recurrence may reach 33% to 50% within five years [6, 7, 8]. Similarly, individual laser modalities such as pulsed dye laser (PDL), carbon dioxide (CO_2_), and erbium: Yttrium aluminum garnet (Er:YAG) systems tend to produce variable outcomes when used alone in this population [8]. These shortcomings highlight the need for more effective therapeutic strategies.

Increasing evidence indicates that combination therapy may compensate for certain limitations of single‐agent treatment. When ablative fractional lasers are combined with corticosteroid delivery, controlled microablation may enhance corticosteroid uptake by creating transepidermal channels that permit greater drug penetration [9]. This laser‐assisted drug delivery pathway may enhance therapeutic effect while allowing lower drug exposure and reducing adverse events, which may be beneficial [10]. In addition, simultaneous targeting of several pathological mechanisms, including vascular proliferation through vascular‐specific lasers, collagen remodeling through ablative lasers, and fibroblast suppression through corticosteroids, may achieve better outcomes than sequential treatment of each process separately [11, 12].

Reliable assessment of scar outcomes requires validated instruments. The Vancouver Scar Scale (VSS) and the Patient and Observer Scar Assessment Scale (POSAS) remain the most widely used tools in scar research, although both have known limitations in certain patient groups [1]. A recent Cochrane systematic review of laser monotherapy found that available evidence was insufficient to confirm superiority over other approaches [8]. Moreover, no comprehensive meta‐analysis has specifically examined laser plus triamcinolone combination therapy against single‐modality treatment using standardized outcome measures, which formed the rationale for this review.

Therefore, we conducted this systematic review and meta‐analysis aiming to integrate evidence from randomized controlled trials (RCTs) and non‐randomized comparative studies that have assessed combined laser and intralesional triamcinolone therapy vs. monotherapy for pathological scars.

## 2 Methods

### 2.1 Study Registration

The study protocol was registered on PROSPERO (CRD420261366585). This systematic review adhered to the Preferred Reporting Items for Systematic Reviews and Meta‐Analyses (PRISMA) 2020 statement standards [13] and was conducted in accordance with the guidelines of the Cochrane handbook for systematic reviews [14]. The PRISMA checklist is provided in the supplementary appendix.

### 2.2 Literature Search and Study Selection

We searched PubMed, Scopus, Web of Science, Embase, and Cochrane Library databases from inception up to March 2026 using these search terms in PubMed: (“Keloid”[Mesh] OR “Cicatrix, Hypertrophic”[Mesh] OR keloid*[tiab] OR “hypertrophic scar*”[tiab] OR “pathologic* scar*”[tiab] OR “post‐burn scar*”[tiab] OR postburn scar*[tiab] OR “burn scar*”[tiab]) AND (“Laser Therapy”[Mesh] OR laser*[tiab] OR “fractional CO2”[tiab] OR “fractional carbon dioxide”[tiab] OR “pulsed dye laser”[tiab] OR PDL[tiab] OR “Er:YAG”[tiab] OR erbium:YAG[tiab] OR “Nd:YAG”[tiab] OR neodymium:YAG[tiab] OR “ablative fractional laser”[tiab] OR “nonablative fractional laser”[tiab] OR “non‐ablative fractional laser”[tiab]) AND (“Triamcinolone”[Mesh] OR triamcinolone[tiab] OR “triamcinolone acetonide”[tiab] OR kenalog[tiab] OR ((intralesional[tiab] OR intradermal[tiab]) AND (corticosteroid*[tiab] OR steroid*[tiab] OR inject*[tiab]))).

No language restrictions were applied at the database level during the search. However, non‐English full texts were excluded during eligibility screening due to the absence of translation resources. Detailed search strategies are displayed in Table S1. References were uploaded to Rayyan for screening [15]. Two independent reviewers conducted a two‐step screening, including title/abstract screening and full‐text screening, to decide on final eligibility. Disagreements were resolved by a third reviewer.

### 2.3 Eligibility Criteria

We included studies that met the following PICO criteria:

- **Population:** Adult patients with pathological scars (Hypertrophic, keloids, post‐burn).
- **Intervention:** Laser therapy (pulsed dye laser, fractional CO2, Er:YAG, or Nd:YAG) combined with intralesional triamcinolone acetonide.
- **Control:** Intralesional triamcinolone monotherapy OR laser monotherapy.
- **Outcomes:** At least one of the following outcomes: Change in VSS/modified Vancouver Scar Scale (mVSS), patient satisfaction, or recurrence rate.
- **Study design:** Randomized controlled trials (RCTs) and non‐randomized studies were included.

We excluded studies not fulfilling these criteria.

### 2.4 Data Extraction

Two independent authors conducted the data extraction using a standardized Excel sheet. The standardized sheet included three domains:

1. Study characteristics, including: Study design, country, allocation method, laser type, triamcinolone regimen, number of sessions, follow‐up duration, and outcome domains reported.
2. Baseline characteristics of the included study population, including: Number of participants, number of scars, age, sex, scar type, etiology, scar duration, previous treatment, and baseline severity.
3. Measurements of the studied outcomes (VSS/mVSS, subscales, satisfaction, recurrence).

### 2.5 Quality Assessment and Certainty of Evidence

Two independent authors evaluated the risk of bias in included RCTs using the Cochrane Risk of Bias two (ROB‐2) assessment tool [16]. This evaluation encompassed an assessment of the randomization process, concealment of the allocation sequence, deviations from the intended interventions, utilization of appropriate analysis to estimate the effect of assignment to intervention, measurement of the outcome, selection of the reported results, and overall risk of bias. Non‐randomized studies were assessed using the ROBINS‐I tool [17]. Any conflict was resolved by a third author. The Robvis web tool was used to create quality assessment figures [18]. The certainty of evidence was evaluated using GRADE assessment [19].

### 2.6 Statistical Analysis

All statistical analyses were performed using R software (version 4.5.0; R Foundation for Statistical Computing, Vienna, Austria) with the meta and metasens packages and R Studio 2024.12.1 + 563 [20, 21]. For continuous outcomes (change in VSS/mVSS scores and patient satisfaction post‐treatment), pooled effect estimates were calculated using the standardized mean difference (SMD), while for dichotomous outcomes, risk ratios (RRs) were used. Hedges' g correction was applied for small sample sizes. Standard deviations were extracted directly from published reports. Pooled estimates were obtained using the inverse‐variance method for continuous data and the Mantel–Haenszel method for dichotomous data. A random‐effects model was used as the primary analytical approach. Between‐study heterogeneity was assessed using the Cochran Q test, I^2^ statistic, and τ^2^, with τ^2^ estimated using the restricted maximum‐likelihood (REML) method. Confidence intervals for τ^2^ and τ were calculated using the Q‐profile method. Heterogeneity was interpreted according to established thresholds (low ≈25%, moderate ≈50%, high ≈75%).

Pre‐specified subgroup analyses were conducted according to the type of comparator (laser monotherapy vs. intralesional triamcinolone monotherapy). Sensitivity analysis excluding non‐randomized high‐risk studies was not feasible because each subgroup contained only two studies; omitting one would have left a single study representing the overall effect, which would not be meaningful. Publication bias assessment was not feasible due to the limited number of included studies [22]. For split‐scar designs, patient‐level data were not available to calculate within‐patient standard deviations; therefore, lesions were treated as independent units in the primary analysis, which may underestimate variance.

## 3 Results

### 3.1 Literature Search

**FIGURE 1:**
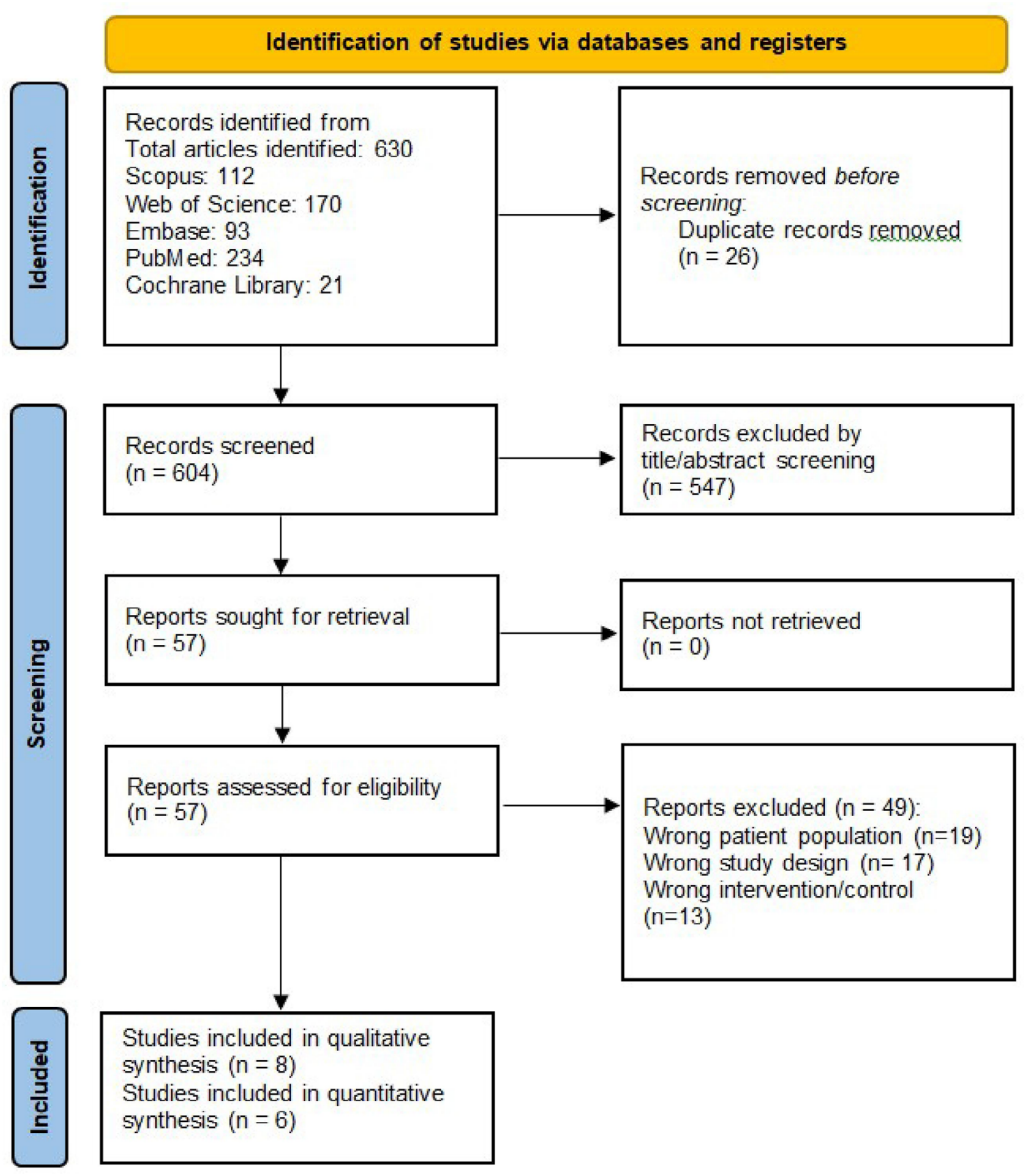
PRISMA flow chart.

Our literature search initially retrieved 630 records; after removing duplicates, 604 studies were screened by title and abstract. Of these, only 57 were reviewed in the full‐text screening stage. Finally, eight studies were included for qualitative synthesis [12, 23, 24, 25, 26, 27, 28, 29], and six studies were included in the quantitative synthesis [12, 23, 25, 26, 27, 29]. The PRISMA flow diagram is shown in Figure 1.

### 3.2 Study Characteristics

A total of 8 studies [12, 23, 24, 25, 26, 27, 28, 29] (5 RCTs, 3 non‐randomized comparative studies) included 320 participants, of whom 248 patients were included in the analysis across six studies [12, 23, 25, 26, 27, 29]. The mean age of the included studies' population ranged from 17.2 to 41.4 years. The percentage of female participants ranged from 28% to 100%. Scar types included hypertrophic scars and keloids from various etiologies (post‐surgical, post‐burn, post‐traumatic) (Table 1 and Table 2, Table S2).

**TABLE 1:** Summary characteristics of the included studies.

Study	Country	Study design	*N* participants	Population	Comparator	Laser	TAC regimen	Sessions	Follow‐up	Outcome domains reported
Alster et al., 2003	USA	Prospective randomized within‐patient controlled trial	22	Hypertrophic inframammary breast‐reduction scars	PDL alone vs. PDL + IL triamcinolone	585‐nm PDL	10–20 mg intralesional triamcinolone immediately after PDL	2 sessions, 6 weeks apart	6 weeks after final treatment	VSS/mVSS, Symptoms, Histology
Alexander et al., 2019	India	Nonrandomized comparative	50	Keloids and hypertrophic scars	Fractional CO2 laser + IL triamcinolone vs. IL triamcinolone alone	Fractional CO2 laser	IL triamcinolone 10 mg/mL	4 sessions	End of fourth session	Dimensions, Satisfaction, Adverse events
Abdel Rahman et al., 2021	Egypt	RCT	45	Hypertrophic scars	Nd:YAG alone vs. Nd:YAG + IL triamcinolone	Long‐pulsed Nd:YAG 1 064 nm	IL triamcinolone 20–40 mg/mL immediately after each laser session	4 sessions, every 4 weeks	3 months after the last session	VSS/mVSS, Symptoms, Adverse events
Shin et al., 2019	Republic of Korea	Nonrandomized comparative	38	Hypertrophic scars and keloids	IL triamcinolone alone vs. NAFL + IL triamcinolone	1 550‐nm erbium‐glass NAFL	1:1 mix of triamcinolone 40 mg/mL + lidocaine 2%; intradermal 0.1 mL/cm^2^	Repeated every 4 weeks (mean 6.95 vs. 5.47 sessions)	6 months	POSAS, Recurrence, Adverse events
Rutnin et al., 2025	Thailand	RCT	25	Post‐mastectomy hypertrophic scars and keloids in transgender men	IL triamcinolone alone vs. 595‐nm PDL + IL triamcinolone	595‐nm PDL	IL triamcinolone 10 mg/mL, 0.3–1 mL/scar after PDL	4 monthly sessions	1, 3, and 6 months after final treatment	VSS/mVSS, Dimensions, Satisfaction, Adverse events, Histology
Park et al., 2025	Republic of Korea	Nonrandomized comparative	111	Postoperative keloids after excision	IL triamcinolone alone vs. IL triamcinolone + Q‐switched Nd:YAG	Q‐switched Nd:YAG	Immediate postoperative TAC plus serial injections of 0.1 mL/cm^2^	5 sessions beginning 3 weeks post‐op, every 4 weeks	6 and 12 months post‐op	VSS/mVSS, POSAS, Satisfaction, Recurrence, Adverse events
Goel et al., 2025	India	RCT	34	Post‐burn hypertrophic scars	Er:YAG alone vs. Er:YAG + intralesional triamcinolone	Er:YAG 2 940 nm	Added to one split‐scar half; dose 0.5–1 mg/kg (maximum reported as 40 mg/dL)	4 sessions, every 4 weeks	2 months after the last session (5th visit)	Dimensions, Symptoms, Satisfaction, Adverse events
Atefi et al., 2025	Iran	RCT	10	Hypertrophic scars and keloids	PDL alone vs. PDL + triamcinolone	PDL 595 nm	PDL followed by triamcinolone 20 mg/cc	3 treatment sessions +1 follow‐up visit	Fourth visit (1 month after last session)	VSS/mVSS, Symptoms, Satisfaction, Adverse events

**TABLE 2:** Baseline characteristics of the participants.

Study	Arm	*N* patients	Age, year, mean±SD	Sex (female), *n* (%)	Scar type baseline	Location/site baseline	Etiology/cause	Scar duration
Alster et al., 2003	PDL alone	22	38 ± 6.3	22 (100%)	Hypertrophic inframammary scars after bilateral breast reduction	Bilateral inframammary scars	Breast reduction surgery	Mean 18.5 months (range 4–72)
	PDL + IL triamcinolone							
Alexander et al., 2019	Fractional CO2 + IL triamcinolone	25	NR	7 (28%)	HTS 8 lesions; keloids 22 lesions; Fitzpatrick IV 4, V 26	Multisite lesions; text extraction captured face 3, forearm 3, hand 3, leg 1, shoulder 1 (additional sites not fully legible)	Overall study: Mostly trauma (69.6%), acne (16.1%), spontaneous (14.3%)	6 months to 20 years; most lesions 1–3 years
	IL triamcinolone alone	25		5 (20%)	HTS 2 lesions; keloids 24 lesions; Fitzpatrick V 26	Multisite lesions; text extraction captured face 2, foot 1, shoulder 2 (additional sites not fully legible)		
Abdel Rahman et al., 2021	Nd:YAG alone	15	17.2 ± 12.7	25 (55.6%)	Hypertrophic scars	Overall study mostly upper limb (38%) and chest (27%); also face, back, lower limb, neck, abdomen	Burns, surgery, or trauma	12.8 ± 8.05 months
	Nd:YAG + IL triamcinolone	15						12.8 ± 6.60 months
Shin et al., 2019	IL triamcinolone alone	21	41.38 ± 11.6	13 (62%)	Hypertrophic scars 12; keloids 9	Head & neck 2; trunk 9; upper extremity 2; lower extremity 3; joint 5	Trauma 9; surgery 12	15.38 months (range 6–23)
	NAFL + IL triamcinolone	17	37.23 ± 10.75	8 (47%)	Hypertrophic scars 8; keloids 9	Head & neck 3; trunk 8; upper extremity 2; lower extremity 1; joint 3	Trauma 8; surgery 9	16.76 months (range 6–24)
Rutnin et al., 2025	IL triamcinolone alone	25	32.8 ± 6.91	0	15 nipple HTS pairs; 12 inframammary HTS pairs; 2 nipple keloid pairs; 6 inframammary keloid pairs	Nipple and inframammary chest scars	Post‐mastectomy scars after top surgery	Median 20 months (IQR 11–57); overall
	595‐nm PDL + IL triamcinolone							
Park et al., 2025	IL triamcinolone alone	58	34.8 ± 25.4	41 (71%)	Postoperative keloids after excision	Ear 28; face 3; chest 9; abdomen 6; back 3; extremity 9	Keloid excision cohort	Time from keloid development to surgery: 6.6 ± 13.7 months
	IL triamcinolone + Q‐switched Nd:YAG	53	35.6 ± 24.7	39 (74%)		Ear 27; face 3; chest 8; abdomen 5; back 3; extremity 7		Time from keloid development to surgery: 6.2 ± 11.9 months
Goel et al., 2025	Er:YAG alone	34	26.03 ± 8.05	26 (76%)	Post‐burn hypertrophic scars	NR	Scald 73.5%, flame 14.7%, contact 8.8%, electrical 2.9%	Mean 3.66 ± 3.17 years (range 1–10)
	Er:YAG + IL triamcinolone							
Atefi et al., 2025	PDL alone	10	36.00 ± 13.23	4 (40%)	Relevant‐arm lesions: Keloid 6, hypertrophic 4	Relevant‐arm lesion sites: Trunk 4, upper limb 3, lower limb 2, neck 1	Hypertrophic scars and keloids	Mean 11.7 months
	PDL + triamcinolone					Relevant‐arm lesion sites: Lower limb 4, trunk 3, upper limb 3		Mean 8.7 months

### 3.3 Quality Assessment and Grading the Certainty of Evidence

**FIGURE 2:**
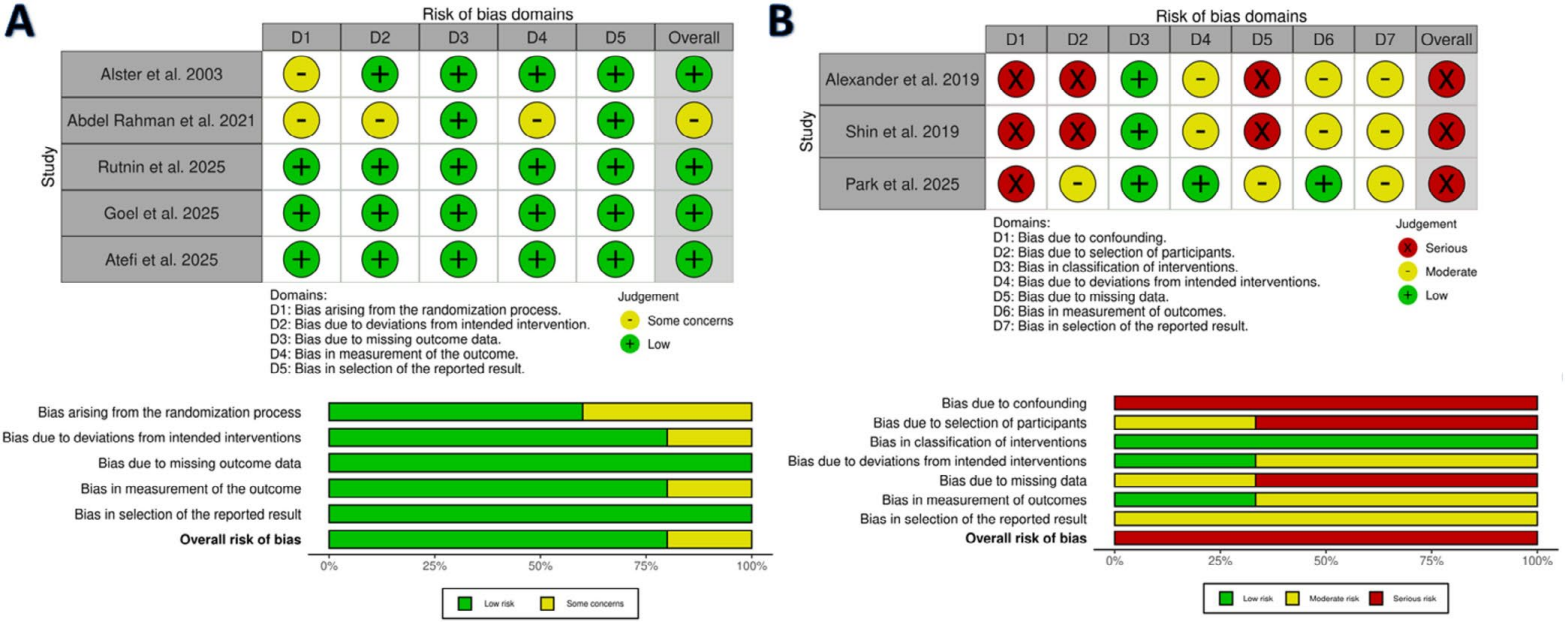
Risk‐of‐bias assessment of the included randomized controlled trials using RoB 2 and non‐randomized studies using ROBINS‐I.

Five original RCTs were evaluated using the ROB2 assessment tool. Four studies (Alster et al., 2003 [24]; Rutnin et al., 2025 [23], Goel et al., 2025 [25], Atefi et al., 2025 [26]) had an overall low risk of bias; one study (Abdel Rahman et al., 2021 [27]) showed some concerns. The three non‐randomized studies (Alexander et al., 2019 [28]; Shin et al., 2019 [12]; Park et al., 2025 [29]) were evaluated using ROBINS‐I, and all showed a serious overall risk of bias due to confounding and selection of participants, as presented in Figure 2. GRADE assessment indicated moderate certainty for the combination therapy over laser monotherapy in improving VSS/mVSS scores and VSS subscales (vascularity, pliability, height, pigmentation). Low certainty was assigned to patient satisfaction and recurrence rate. Very low certainty was found for comparisons against intralesional triamcinolone alone for VSS/mVSS change due to inclusion of non‐randomized studies with serious risk of bias, substantial heterogeneity, and imprecision (Table 3).

**TABLE 3:** GRADE assessment.

Outcome	Number of studies	Study design	Risk of bias	Inconsistency	Indirectness	Imprecision	Other considerations	Effect estimate (95% CI)	Certainty
VSS/mVSS change – vs. IL triamcinolone alone	2	RCTs + non‐RCT	Serious^a^	Serious (I^2^ = 83.1%)^b^	Not serious	Serious^c^	None	SMD 0.28 (−0.53 to 1.10)	⨁◯◯◯ **Very Low**
VSS/mVSS change – vs. laser monotherapy	2	RCTs	Not serious^d^	Not serious (I^2^ = 16.9%)	Not serious	Serious^e^	None	SMD −1.16 (−1.85 to −0.48)	⨁⨁⨁◯ **Moderate**
VSS change – Vascularity (vs. laser)	2	RCTs	Not serious^d^	Not serious (I^2^ = 6.2%)	Not serious	Serious^e^	None	SMD −1.06 (−1.68 to −0.43)	⨁⨁⨁◯ **Moderate**
VSS change – Pliability (vs. laser)	2	RCTs	Not serious^d^	Not serious (I^2^ = 0%)	Not serious	Serious^e^	None	SMD −1.07 (−1.68 to −0.47)	⨁⨁⨁◯ **Moderate**
VSS change – Pigmentation (vs. laser)	2	RCTs	Not serious^d^	Not serious (I^2^ = 0%)	Not serious	Serious^f^	None	SMD −0.12 (−0.68 to 0.43)	⨁⨁⨁◯ **Moderate**
VSS change – Height (vs. laser)	2	RCTs	Not serious^d^	Not serious (I^2^ = 0%)	Not serious	Serious^e^	None	SMD −0.89 (−1.47 to −0.30)	⨁⨁⨁◯ **Moderate**
Patient satisfaction – vs. IL triamcinolone alone	2	RCTs + Non‐RCT	Serious^a^	Not serious (I^2^ = 0%)	Not serious	Serious^e^	None	SMD 0.33 (0.01 to 0.64)	⨁⨁◯◯ **Low**
Patient satisfaction – vs. laser monotherapy	1	RCT	Not serious^d^	Not applicable	Not serious	Serious^g^	None	SMD 1.38 (0.85 to 1.91)	⨁⨁◯◯ **Low**
Recurrence rate (vs. IL triamcinolone alone)	2	Non‐RCT	Serious^a^	Not serious (I^2^ = 27.3%)	Not serious	Serious^h^	None	RR 0.65 (0.16 to 2.67)	⨁⨁◯◯ **Low**

^a^ Notes for downgrading: Risk of Bias: Serious: This comparison includes non‐randomized evidence assessed as having a serious overall risk of bias. The presence of non‐randomized studies with serious bias warrants downgrading by one level.

^b^ Inconsistency: Serious: Substantial heterogeneity was observed (I^2^ = 83.1%). Downgraded by one level.

^c^ Imprecision: Serious: The 95% confidence interval crosses the null (−0.53 to 1.10) and is wide, indicating no statistically significant effect. The total sample size is modest. Downgraded by one level.

^d^ Risk of Bias: Not serious: All studies contributing to these comparisons were RCTs with an overall low/some concerns risk of bias according to ROB2 assessment. No downgrading applied.

^e^ Imprecision: Serious: Although the effect estimates are statistically significant and the confidence intervals do not cross the null, only 2 studies contributed to each of these analyses, and the total sample size is modest. Downgraded by one level.

^f^ Imprecision: Serious: The 95% confidence interval crosses the null (−0.68 to 0.43), indicating no statistically significant effect. Additionally, only 2 studies with a modest sample size contributed to this analysis. Downgraded by one level.

^g^ Imprecision: Serious: This estimate is derived from a single study with a modest sample size. Downgraded by one level.

^h^ Imprecision: Serious: The 95% confidence interval is very wide (0.16 to 2.67), crosses the null, and the total number of events is small. Only 2 studies with modest sample sizes contributed. Downgraded by one level.

### 3.4 Outcomes

#### 3.4.1 Primary Outcomes

##### 3.4.1.1 Change in VSS/mVSS

**FIGURE 3:**
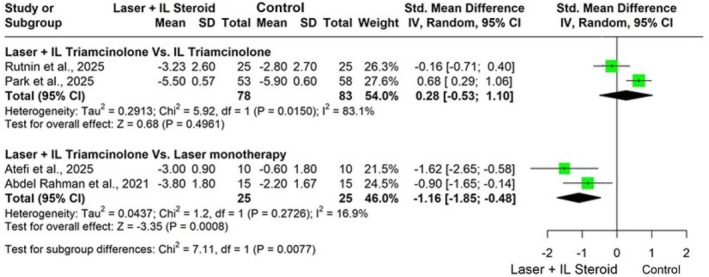
Forest plot of the change in Vancouver Scar Scale/modified Vancouver Scar Scale (VSS/mVSS) comparing laser plus intralesional (IL) triamcinolone with intralesional (IL) triamcinolone and laser monotherapy.

Compared with intralesional triamcinolone alone, the combination therapy did not demonstrate a significant benefit (SMD = 0.28, 95% CI: −0.53 to 1.10, *p* = 0.496), and heterogeneity was considerable (I^2^ = 83.1%). In contrast, when compared with laser monotherapy, laser plus intralesional triamcinolone showed a significant reduction in VSS/mVSS scores (SMD = −1.16, 95% CI: −1.85 to −0.48, *p* = 0.0008), with low heterogeneity (I^2^ = 16.9%). There was a statistically significant difference between subgroups (*p* = 0.0077), indicating that the effect of combination therapy varies depending on the comparator, with clear benefit over laser alone but not over intralesional triamcinolone monotherapy (Figure 3).

##### 3.4.1.2 Change in VSS Subscales

**FIGURE 4:**
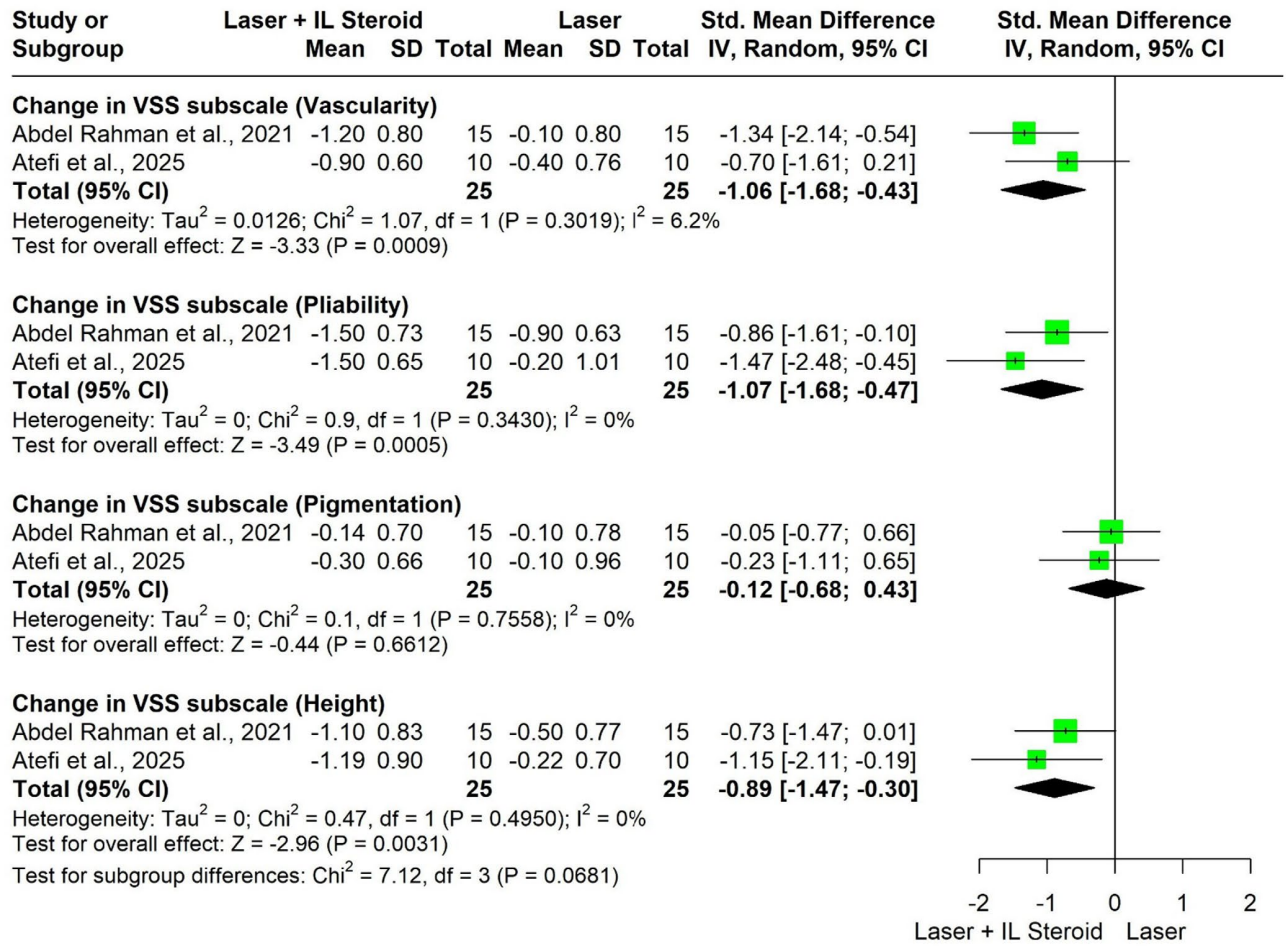
Forest plot comparing laser plus intralesional triamcinolone with laser monotherapy for changes in VSS/mVSS subscales.

When compared with laser monotherapy, laser combined with intralesional triamcinolone was associated with significant improvements in vascularity (SMD = −1.06, 95% CI: −1.68 to −0.43, *p* = 0.0009) with low heterogeneity (I^2^ = 6.2%), and in pliability (SMD = −1.07, 95% CI: −1.68 to −0.47, *p* = 0.0005) with no observed heterogeneity (I^2^ = 0%). Similarly, scar height showed a significant improvement favoring the combination therapy (SMD = −0.89, 95% CI: −1.47 to −0.30, *p* = 0.0031), with no heterogeneity (I^2^ = 0%). In contrast, no significant difference was observed for pigmentation (SMD = −0.12, 95% CI: −0.68 to 0.43, *p* = 0.661), with negligible heterogeneity (I^2^ = 0%). The test for subgroup differences was not statistically significant (*p* = 0.068), suggesting that although improvements were evident in specific domains (vascularity, pliability, and height), the magnitude of effect did not differ significantly across subscales (Figure 4).

#### 3.4.2 Secondary Outcomes

##### 3.4.2.1 Patient Satisfaction

**FIGURE 5:**
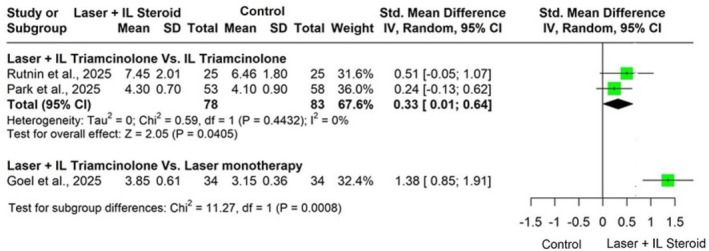
Forest plot of patient satisfaction comparing laser plus intralesional (IL) triamcinolone with intralesional (IL) triamcinolone and laser monotherapy.

Satisfaction scales included visual analogue scales (0–10) in [23] and Likert scales [1, 2, 3, 4, 5] in the other two studies, with higher scores indicating better satisfaction. All scales were patient‐reported and validated. SMD was used because different scales were employed across studies. In subgroup analysis, when compared with intralesional triamcinolone alone, combination therapy showed a modest but statistically significant improvement in patient satisfaction (SMD = 0.33, 95% CI: 0.01 to 0.64, *p* = 0.040), with no heterogeneity (I^2^ = 0%). In contrast, a larger effect size was observed when compared with laser monotherapy (SMD = 1.38, 95% CI: 0.85 to 1.91), based on a single study. The finding for laser monotherapy comparison is based on a single study and should be interpreted cautiously. The test for subgroup differences was statistically significant (*p* = 0.0008), indicating that the magnitude of improvement in patient satisfaction differed significantly depending on the comparator, with greater benefit observed against laser monotherapy than intralesional triamcinolone alone (Figure 5).

##### 3.4.2.2 Recurrence Rate

**FIGURE 6:**
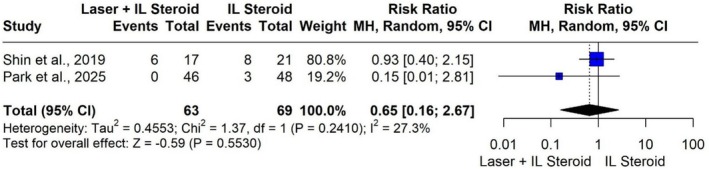
Forest plot comparing laser plus intralesional triamcinolone with intralesional triamcinolone alone for recurrence rate.

Evidence remains insufficient to determine whether recurrence rates differ between combination therapy and intralesional triamcinolone alone (RR = 0.65, 95% CI 0.16 to 2.67, *p* = 0.553; I^2^ = 27.3%) (Figure 6).

## 4 Discussion

### 4.1 Summary of Findings

Laser plus intralesional triamcinolone did not show a statistically significant difference in reducing VSS/mVSS or recurrence rate compared with intralesional triamcinolone monotherapy. However, when laser combined with intralesional triamcinolone was directly compared against laser monotherapy, the combination demonstrated superiority in reducing scar scores, with an SMD of −1.16, favoring combination therapy. Regarding VSS subscales, vascularity, pliability, and scar height showed a significant reduction in favor of combination therapy. In addition, our findings demonstrated that patient satisfaction was significantly higher with combination therapy.

### 4.2 Interpretation of Findings

The lack of a clear advantage for combination therapy over intralesional triamcinolone monotherapy should be interpreted with caution in the context of the broader literature. A recent network meta‐analysis reported that triamcinolone combined with 5‐fluorouracil produced the most consistent gains in treatment response and recurrence prevention among intralesional regimens [30]. This pattern suggests that adding laser therapy to triamcinolone may not confer the same benefit as pairing triamcinolone with another active drug. However, this is a hypothesis based on indirect comparisons rather than direct evidence from the present meta‐analysis. Direct head‐to‐head trials are needed to determine whether pharmacological combinations offer advantages over laser‐assisted drug delivery.

The superiority of combination therapy over laser monotherapy with moderate certainty of evidence is in line with the broader evidence favoring multimodal treatment. Prior studies have repeatedly shown that single‐modality approaches tend to produce less reliable outcomes than combined strategies [30, 31]. The more effective performance of laser plus triamcinolone compared with laser alone indicates that the steroid component contributes meaningfully to scar remodeling. These findings indicate that simultaneous targeting of multiple pathogenic pathways may enhance treatment response. In the comparison of laser monotherapy vs. combination therapy, heterogeneity was low (I^2^ = 16.9%), which supports the reliability of our reported result and suggests that the included studies yielded largely consistent outcomes.

The subscale results add further detail to the effect of this regimen with moderate certainty of evidence. Compared with laser alone, combination therapy significantly improved vascularity and pliability, with low heterogeneity in either analysis. These results suggest that triamcinolone addresses aspects of scar biology that laser treatment may not fully correct. Intralesional corticosteroids reduce inflammation and suppress collagen deposition, which differs from the thermal remodeling induced by laser [32, 33, 34]. Scar height also improved significantly with combination treatment, likely reflecting the additive effect of pharmacological and physical interventions. By contrast, pigmentation did not improve significantly in any group, implying that neither laser nor triamcinolone adequately corrects dyschromia and that other targeted therapies may be needed for this component of scar pathology.

Patient satisfaction is an especially important outcome, yet it is often underweighted in scar studies. In our analysis, combination therapy was associated with greater satisfaction, suggesting that patients perceived a broader overall benefit that incorporated appearance, symptoms, and function (low certainty of evidence). Bernabe et al. likewise found that combination regimens generally yielded better patient satisfaction than monotherapy across scar treatments [31]. This emphasis on patient‐reported benefit is consistent with current clinical practice, where subjective outcomes are increasingly central to treatment selection, especially in these patients.

The evidence on triamcinolone plus 5‐fluorouracil more consistently shows lower recurrence than triamcinolone alone [35, 36]. The limited effect of laser on recurrence may reflect the fact that laser‐induced tissue changes do not provide the sustained antifibrotic action achieved by pharmacological agents. Recent evidence suggests that combinations targeting specific inflammatory and fibrotic pathways, such as triamcinolone with 5‐fluorouracil, may be more effective for long‐term control than pairing a drug with a mechanical modality such as laser [37, 38]. This implies that pharmacological synergy may be more important than combining mechanical and pharmacological interventions when the goal is recurrence prevention. However, the evidence has low certainty and is insufficient to draw definitive conclusions.

Wang et al. reported consistent findings. They found long‐term benefit with ultrapulse fractional CO2 laser plus topical triamcinolone in refractory keloids; that study involved a selected population, a specialized protocol, and intensive follow‐up, which limits direct generalization [39]. Likewise, more recent systematic studies on fractional CO2 plus triamcinolone in hypertrophic scars suggest promising improvements, but it also shows that results vary by scar subtype, treatment sequence, and follow‐up length [40, 41, 42]. These findings suggest that laser‐steroid therapy is useful, but it is not interchangeable with pharmacological combinations. Its best role is probably as a targeted adjunct for scars that remain vascular, firm, or symptomatic after standard injection therapy, rather than as a default option for every case.

### 4.3 Strengths and Limitations

To our knowledge, our meta‐analysis is the first to directly compare intralesional triamcinolone plus laser therapy with monotherapy approaches. We followed PRISMA guidelines and used a robust analytical framework for this meta‐analysis, with random‐effects modeling and conventional heterogeneity testing based on Cochran's Q, I^2^, and tau‐squared estimated by restricted maximum likelihood. In addition, prespecified subgroup analyses by follow‐up duration made it possible to examine whether treatment effects changed over time. Together, these methodological features strengthen confidence in the findings.

Nonetheless, several limitations should be acknowledged. The presence of substantial heterogeneity in a number of comparisons makes it difficult to draw firm conclusions about the relative superiority of the interventions in these cases. Between‐study heterogeneity may reflect differences in the laser systems used, such as fractional carbon dioxide, pulsed dye, and erbium‐glass lasers, which differ in wavelength and depth of tissue penetration. Variation in treatment settings, including fluence, pulse duration, and interval between sessions, was also substantial across the included studies, which limits the extent to which the results can be generalized. Moreover, differences in skin phototype, scar site, patient age, and scar maturity can meaningfully affect treatment response, and they may explain the variability reported between studies. The pooled estimates may have been further affected by inconsistency in triamcinolone dosing, which ranged from 20 to 40 mg/mL, as well as by differences in injection volume. Follow‐up data beyond 12 months were limited, so the durability of the benefit and the risk of late recurrence could not be assessed with confidence. Furthermore, scar etiology was not thoroughly reported; hence, it was not possible to determine whether the effect of combination therapy differs between burn‐related scars and post‐surgical scars, which are known to heal through different biological pathways [43]. The comparison against intralesional triamcinolone alone includes non‐randomized evidence with serious risk of bias; however, sensitivity analysis was not feasible due to the limited number of included studies. For split‐scar designs, patient‐level data were not available to calculate within‐patient standard deviations, which may underestimate variance.

### 4.4 Clinical Implications and Future Recommendations

These findings support laser‐assisted drug delivery as a practical treatment option. However, the evidence does not support superiority over intralesional triamcinolone alone, and the recurrence benefit remains uncertain. The primary advantage of combination therapy appears to be over laser monotherapy rather than over steroid injection alone. The findings also suggest that pharmacological combinations may offer an alternative approach that warrants further investigation. In patients with established hypertrophic scars in the absence of a strong history of recurrence, laser combined with intralesional triamcinolone appears to improve scar‐specific outcomes and patient satisfaction more than laser monotherapy. By contrast, for keloids with a high risk of recurrence, the available evidence suggests that intralesional triamcinolone combined with 5‐fluorouracil may provide better long‐term control [44]. Treatment decisions should therefore be tailored to scar type, recurrence risk, patient preference, skin phototype, and lesion site. Future studies should adopt more uniform treatment protocols so that findings can be compared more consistently across trials. Head‐to‐head randomized controlled trials of laser plus triamcinolone vs. triamcinolone plus 5‐fluorouracil are needed to determine whether mechanical or drug‐based synergy offers greater benefit. In addition, patient‐reported outcomes should be incorporated more regularly through validated measures that reflect both perceived benefit and objective scar change. Further research in individuals with darker skin types is also important since hypertrophic scars and keloids are more common in these populations [45]. Longer follow‐up, ideally beyond 12 months, is needed to determine the durability of response and to define appropriate retreatment intervals.

## 5 Conclusion

This meta‐analysis suggests that laser therapy combined with intralesional triamcinolone acetonide may be more effective than laser monotherapy for improving scar vascularity, pliability, and height. However, current evidence is insufficient to demonstrate superiority over intralesional triamcinolone alone, and the recurrence benefit remains uncertain. The considerable heterogeneity across studies, together with only modest effects on recurrence, indicates that this combined approach should be viewed as one reasonable option among several rather than a consistently superior treatment. Also, improved patient satisfaction might support the case for clinical use, especially when laser monotherapy is unlikely to achieve adequate outcomes. Finally, future investigations should include head‐to‐head comparisons of combination regimens and longer follow‐up to guide more personalized scar management.

## Author Contributions

Conceptualization: A.M.A., W.A.; Methodology: B.N.A., K.A., N.A., F.F.A., S.A.G., D.J.H., A.J.A., F.A.A., M.A.A., A.H.A., S.A., A.M.A.; Formal analysis: A.M.A.; Data curation: B.N.A., K.A., N.A., F.F.A., S.A.G., D.J.H., A.J.A., F.A.A., M.A.A., A.H.A., S.A.; Investigation: B.N.A., K.A., N.A., F.F.A., S.A.G., D.J.H., A.J.A., F.A.A., M.A.A., A.H.A., S.A.; Software: A.M.A.; Validation: A.M.A.; Supervision: A.M.A., W.A.; Project administration: A.M.A., W.A.; Writing – original draft preparation: B.N.A., K.A., A.M.A.; Writing – review and editing: All authors. All authors have read and agreed to the published version of the manuscript. #Co‐first authors: B.N.A. and K.A. contributed equally to this work.

## Funding

The authors have nothing to report.

## Conflicts of Interest

The authors declare no conflicts of interest.

## Supporting information


**Table S1:** Search strategies.
**DATA S1:** PRISMA checklist.
**Table S2:** Baseline of included studies (continued).

## Data Availability

Data sharing not applicable to this article as no datasets were generated or analysed during the current study.
