# Supplementary material for: Efficacy of Laser‐Assisted Intralesional Triamcinolone for Pathological Scars: A Systematic Review and Meta‐Analysis
Source: J Cosmet Dermatol. 2026 Aug 17;25(8):e71064. doi: 10.1111/jocd.71064 (PMC13479049; doi:10.1111/jocd.71064)
Supplement: Supplementary file 1 [file JOCD-25-e71064-s001.docx]

**Supplementary Appendix**

**Table of Contents**

| Page | Item |
| --- | --- |
| 2-3 | Table S1. Search strategies |
| 4-5 | PRISMA Checklist |
| 6-7 | Table S2: Baseline of included studies (continued) |

Table S1. Search strategies

| Databases | Search strategies | Results | Limitations | Date of search |
| --- | --- | --- | --- | --- |
| PubMed | ( "Keloid"[Mesh] OR "Cicatrix, Hypertrophic"[Mesh] OR keloid*[tiab] OR "hypertrophic scar*"[tiab] OR "pathologic* scar*"[tiab] OR "post-burn scar*"[tiab] OR postburn scar*[tiab] OR "burn scar*"[tiab] ) AND ( "Laser Therapy"[Mesh] OR laser*[tiab] OR "fractional CO2"[tiab] OR "fractional carbon dioxide"[tiab] OR "pulsed dye laser"[tiab] OR PDL[tiab] OR "Er:YAG"[tiab] OR erbium:YAG[tiab] OR "Nd:YAG"[tiab] OR neodymium:YAG[tiab] OR "ablative fractional laser"[tiab] OR "nonablative fractional laser"[tiab] OR "non-ablative fractional laser"[tiab] ) AND ( "Triamcinolone"[Mesh] OR triamcinolone[tiab] OR "triamcinolone acetonide"[tiab] OR kenalog[tiab] OR ( (intralesional[tiab] OR intradermal[tiab]) AND (corticosteroid*[tiab] OR steroid*[tiab] OR inject*[tiab]) ) ) | 234 | No limitations applied | 13 March 2026 |
| WOS | TS=( ( keloid* OR "hypertrophic scar*" OR "pathologic* scar*" OR "post-burn scar*" OR postburn scar* OR "burn scar*" ) AND ( laser* OR "fractional CO2" OR "fractional carbon dioxide" OR "pulsed dye laser" OR PDL OR "Er:YAG" OR "erbium:YAG" OR "Nd:YAG" OR "neodymium:YAG" OR "ablative fractional laser" OR "nonablative fractional laser" OR "non-ablative fractional laser" ) AND ( triamcinolone OR "triamcinolone acetonide" OR kenalog OR ((intralesional OR intradermal) NEAR/3 (corticosteroid* OR steroid* OR inject*)) ) ) | 170 | No limitations applied | 13 March 2026 |
| Cochrane CENTRAL | ( [mh Keloid] OR [mh "Cicatrix, Hypertrophic"] OR keloid*:ti,ab,kw OR "hypertrophic scar*":ti,ab,kw OR "pathologic* scar*":ti,ab,kw OR "post-burn scar*":ti,ab,kw OR postburn scar*:ti,ab,kw OR "burn scar*":ti,ab,kw ) AND ( [mh "Laser Therapy"] OR laser*:ti,ab,kw OR "fractional CO2":ti,ab,kw OR "fractional carbon dioxide":ti,ab,kw OR "pulsed dye laser":ti,ab,kw OR PDL:ti,ab,kw OR "Er:YAG":ti,ab,kw OR "erbium:YAG":ti,ab,kw OR "Nd:YAG":ti,ab,kw OR "neodymium:YAG":ti,ab,kw ) AND ( [mh Triamcinolone] OR triamcinolone:ti,ab,kw OR "triamcinolone acetonide":ti,ab,kw OR kenalog:ti,ab,kw OR ( (intralesional:ti,ab,kw OR intradermal:ti,ab,kw) AND (corticosteroid*:ti,ab,kw OR steroid*:ti,ab,kw OR inject*:ti,ab,kw) ) ) | 21 | Title Abstract Keyword | 13 March 2026 |
| Scopus | TITLE-ABS-KEY( ( keloid* OR "hypertrophic scar*" OR "pathologic* scar*" OR "post-burn scar*" OR postburn scar* OR "burn scar*" ) AND ( laser* OR "fractional CO2" OR "fractional carbon dioxide" OR "pulsed dye laser" OR PDL OR "Er:YAG" OR "erbium:YAG" OR "Nd:YAG" OR "neodymium:YAG" OR "ablative fractional laser" OR "nonablative fractional laser" OR "non-ablative fractional laser" ) AND ( triamcinolone OR "triamcinolone acetonide" OR kenalog OR ((intralesional OR intradermal) W/3 (corticosteroid* OR steroid* OR inject*)) ) ) | 112 | title, Abstract, Keywords | 13 March 2026 |
| Embase | ( 'keloid'/exp OR 'hypertrophic scar'/exp OR keloid*:ti,ab,kw OR 'hypertrophic scar*':ti,ab,kw OR 'pathologic* scar*':ti,ab,kw OR 'post-burn scar*':ti,ab,kw OR postburn scar*:ti,ab,kw OR 'burn scar*':ti,ab,kw ) AND ( 'laser therapy'/exp OR laser*:ti,ab,kw OR 'fractional co2':ti,ab,kw OR 'fractional carbon dioxide':ti,ab,kw OR 'pulsed dye laser':ti,ab,kw OR pdl:ti,ab,kw OR 'er:yag':ti,ab,kw OR 'erbium:yag':ti,ab,kw OR 'nd:yag':ti,ab,kw OR 'neodymium:yag':ti,ab,kw OR 'ablative fractional laser':ti,ab,kw OR 'nonablative fractional laser':ti,ab,kw OR 'non-ablative fractional laser':ti,ab,kw ) AND ( 'triamcinolone'/exp OR triamcinolone:ti,ab,kw OR 'triamcinolone acetonide':ti,ab,kw OR kenalog:ti,ab,kw OR ( (intralesional:ti,ab,kw OR intradermal:ti,ab,kw) AND (corticosteroid*:ti,ab,kw OR steroid*:ti,ab,kw OR inject*:ti,ab,kw) ) ) | 93 | No limitations applied | 13 March 2026 |
| Total |  | 630 |  |  |

**PRISMA checklist**

| **Section and Topic** | **Item #** | **Checklist item** | **Location where item is reported** |
| --- | --- | --- | --- |
| **TITLE** | | |  |
| Title | 1 | Identify the report as a systematic review. | 1 |
| **ABSTRACT** | | |  |
| Abstract | 2 | See the PRISMA 2020 for Abstracts checklist. | 3 |
| **INTRODUCTION** | | |  |
| Rationale | 3 | Describe the rationale for the review in the context of existing knowledge. | 4 |
| Objectives | 4 | Provide an explicit statement of the objective(s) or question(s) the review addresses. | 4 |
| **METHODS** | | |  |
| Eligibility criteria | 5 | Specify the inclusion and exclusion criteria for the review and how studies were grouped for the syntheses. | 5 |
| Information sources | 6 | Specify all databases, registers, websites, organisations, reference lists and other sources searched or consulted to identify studies. Specify the date when each source was last searched or consulted. | 5 |
| Search strategy | 7 | Present the full search strategies for all databases, registers and websites, including any filters and limits used. | 5 |
| Selection process | 8 | Specify the methods used to decide whether a study met the inclusion criteria of the review, including how many reviewers screened each record and each report retrieved, whether they worked independently, and if applicable, details of automation tools used in the process. | 5 |
| Data collection process | 9 | Specify the methods used to collect data from reports, including how many reviewers collected data from each report, whether they worked independently, any processes for obtaining or confirming data from study investigators, and if applicable, details of automation tools used in the process. | 5-6 |
| Data items | 10a | List and define all outcomes for which data were sought. Specify whether all results that were compatible with each outcome domain in each study were sought (e.g. for all measures, time points, analyses), and if not, the methods used to decide which results to collect. | 5 |
|  | 10b | List and define all other variables for which data were sought (e.g. participant and intervention characteristics, funding sources). Describe any assumptions made about any missing or unclear information. | 5-6 |
| Study risk of bias assessment | 11 | Specify the methods used to assess risk of bias in the included studies, including details of the tool(s) used, how many reviewers assessed each study and whether they worked independently, and if applicable, details of automation tools used in the process. | 6 |
| Effect measures | 12 | Specify for each outcome the effect measure(s) (e.g. risk ratio, mean difference) used in the synthesis or presentation of results. | 6 |
| Synthesis methods | 13a | Describe the processes used to decide which studies were eligible for each synthesis (e.g. tabulating the study intervention characteristics and comparing against the planned groups for each synthesis (item #5)). | 5-6 |
|  | 13b | Describe any methods required to prepare the data for presentation or synthesis, such as handling of missing summary statistics, or data conversions. | 6 |
|  | 13c | Describe any methods used to tabulate or visually display results of individual studies and syntheses. | 6-8 |
|  | 13d | Describe any methods used to synthesize results and provide a rationale for the choice(s). If meta-analysis was performed, describe the model(s), method(s) to identify the presence and extent of statistical heterogeneity, and software package(s) used. | 6 |
|  | 13e | Describe any methods used to explore possible causes of heterogeneity among study results (e.g. subgroup analysis, meta-regression). | 6 |
|  | 13f | Describe any sensitivity analyses conducted to assess robustness of the synthesized results. | 6 |
| Reporting bias assessment | 14 | Describe any methods used to assess risk of bias due to missing results in a synthesis (arising from reporting biases). | 6 |
| Certainty assessment | 15 | Describe any methods used to assess certainty (or confidence) in the body of evidence for an outcome. | 6 |
| **RESULTS** | | |  |
| Study selection | 16a | Describe the results of the search and selection process, from the number of records identified in the search to the number of studies included in the review, ideally using a flow diagram. | 6 |
|  | 16b | Cite studies that might appear to meet the inclusion criteria, but which were excluded, and explain why they were excluded. |  |
| Study characteristics | 17 | Cite each included study and present its characteristics. | 7 |
| Risk of bias in studies | 18 | Present assessments of risk of bias for each included study. | 7 |
| Results of individual studies | 19 | For all outcomes, present, for each study: (a) summary statistics for each group (where appropriate) and (b) an effect estimate and its precision (e.g. confidence/credible interval), ideally using structured tables or plots. | 7-8 |
| Results of syntheses | 20a | For each synthesis, briefly summarise the characteristics and risk of bias among contributing studies. | 7-8 |
|  | 20b | Present results of all statistical syntheses conducted. If meta-analysis was done, present for each the summary estimate and its precision (e.g. confidence/credible interval) and measures of statistical heterogeneity. If comparing groups, describe the direction of the effect. | 7-8 |
|  | 20c | Present results of all investigations of possible causes of heterogeneity among study results. | 7-8 |
|  | 20d | Present results of all sensitivity analyses conducted to assess the robustness of the synthesized results. |  |
| Reporting biases | 21 | Present assessments of risk of bias due to missing results (arising from reporting biases) for each synthesis assessed. |  |
| Certainty of evidence | 22 | Present assessments of certainty (or confidence) in the body of evidence for each outcome assessed. | 7 |
| **DISCUSSION** | | |  |
| Discussion | 23a | Provide a general interpretation of the results in the context of other evidence. | 8-10 |
|  | 23b | Discuss any limitations of the evidence included in the review. | 10 |
|  | 23c | Discuss any limitations of the review processes used. | 10 |
|  | 23d | Discuss implications of the results for practice, policy, and future research. | 10-11 |
| **OTHER INFORMATION** | | |  |
| Registration and protocol | 24a | Provide registration information for the review, including register name and registration number, or state that the review was not registered. | 5 |
|  | 24b | Indicate where the review protocol can be accessed, or state that a protocol was not prepared. | 5 |
|  | 24c | Describe and explain any amendments to information provided at registration or in the protocol. | 5 |
| Support | 25 | Describe sources of financial or non-financial support for the review, and the role of the funders or sponsors in the review. | 11 |
| Competing interests | 26 | Declare any competing interests of review authors. | 11 |
| Availability of data, code and other materials | 27 | Report which of the following are publicly available and where they can be found: template data collection forms; data extracted from included studies; data used for all analyses; analytic code; any other materials used in the review. | 11 |

Table S2: Baseline of included studies (continued)

| Study | Arm | N scars/lesions | participant profile | Scar type baseline | Previous treatment baseline | Baseline severity / scale |
| --- | --- | --- | --- | --- | --- | --- |
| Alster et al., 2003 | PDL alone | 22 | skin phototypes I–IV | Hypertrophic inframammary scars after bilateral breast reduction | No scar treatment within 6 months of entry | NR |
|  | PDL + IL triamcinolone | 22 |  |  |  |  |
| Alexander et al., 2019 | Fractional CO2 + IL triamcinolone | 30 | SES LC 8 / MC 11 / UC 6 | HTS 8 lesions; keloids 22 lesions; Fitzpatrick IV 4, V 26 | Topical 2; prior IL steroid 9; cryotherapy 5; laser 1; surgery 1 | Pretreatment dimensions reported lesion-wise; no arm-level summary mean printed |
|  | IL triamcinolone alone | 26 | SES LC 13 / MC 11 / UC 1 | HTS 2 lesions; keloids 24 lesions; Fitzpatrick V 26 | Topical 2; prior IL steroid 1; cryotherapy 0; laser 4; surgery 0 |  |
| Abdel Rahman et al., 2021 | Nd:YAG alone | 15 | NR | Hypertrophic scars | No previous clinical intervention | VSS total 6.5 ± 1.6; vascularity 1.2 ± 0.78; pliability 2.5 ± 0.52; pigmentation 1.0 ± 0.66; height 1.7 ± 0.48 |
|  | Nd:YAG + IL triamcinolone | 15 |  |  |  | VSS total 8.0 ± 1.56; vascularity 2.1 ± 0.73; pliability 2.7 ± 0.67; pigmentation 0.8 ± 0.60; height 2.4 ± 0.59 |
| Shin et al., 2019 | IL triamcinolone alone | 21 | NR | Hypertrophic scars 12; keloids 9 | NR | POSAS patient 38.38; observer 42.08; total 80.46 |
|  | NAFL + IL triamcinolone | 17 | NR | Hypertrophic scars 8; keloids 9 |  | POSAS patient 36.18; observer 38.18; total 74.36 |
| Rutnin et al., 2025 | IL triamcinolone alone | 35 | skin type III 7 (28%), IV 18 (72%); all on testosterone therapy | 15 nipple HTS pairs; 12 inframammary HTS pairs; 2 nipple keloid pairs; 6 inframammary keloid pairs | Overall: IL TAC 11; IL TAC + silicone sheet 1; silicone gel sheet 2; topical silicone gel 4 | VSS 7.314 ± 2.36 |
|  | 595-nm PDL + IL triamcinolone | 35 |  |  |  | VSS 7.457 ± 2.29 |
| Park et al., 2025 | IL triamcinolone alone | 58 | All East Asian participants | Postoperative keloids after excision | Prior TAC injection 44; surgery 11; laser 2; radiotherapy 1; chemotherapy agent 0 | mVSS 9.3 ± 0.6; POSAS 7.9 ± 1.4 |
|  | IL triamcinolone + Q-switched Nd:YAG | 53 | all East Asian participants |  | Prior TAC injection 45; surgery 7; laser 1; radiotherapy 0; chemotherapy agent 0 | mVSS 9.1 ± 0.5; POSAS 8.2 ± 1.7 |
| Goel et al., 2025 | Er:YAG alone | 34 | Fitzpatrick type V 79.4%, type IV 20.6% | Post-burn hypertrophic scars | NR | UNC4P baseline—Pain: Score0 20, Score1 12, Score2 2; Pruritus: Score0 7, Score1 18, Score2 9; Paraesthesia: Score0 12, Score1 19, Score2 3; Pliability: Score0 7, Score1 14, Score2 13 |
|  | Er:YAG + IL triamcinolone | 34 |  |  |  |  |
| Atefi et al., 2025 | PDL alone | 10 | NR | Relevant-arm lesions: keloid 6, hypertrophic 4 | clobetasol ointment, CO2 laser, or none | Total VSS 7.90 ± 1.52; vascularity 2.00 ± 0.67; pigmentation 0.60 ± 0.96; pliability 3.20 ± 0.91; height 2.10 ± 0.73 |
|  | PDL + triamcinolone | 10 |  |  |  | Total VSS 7.30 ± 0.24; vascularity 1.90 ± 0.60; pigmentation 0.50 ± 0.60; pliability 3.70 ± 0.32; height 2.09 ± 0.92 |
